# Isolation and identification of fungi associated with spoilt fruits vended in Gwagwalada market, Abuja, Nigeria

**DOI:** 10.14202/vetworld.2017.393-397

**Published:** 2017-04-10

**Authors:** Samuel Mailafia, God’spower Richard Okoh, Hamza Olatunde K. Olabode, Ramatu Osanupin

**Affiliations:** 1 Department of Veterinary Microbiology, Faculty of Veterinary Medicine, University of Abuja, PMB 117, Abuja, Nigeria; 2 Department of Biological Sciences, Faculty of Sciences, University of Abuja, PMB 117, Abuja, Nigeria

**Keywords:** frequency of occurrence, fruits, fungi, pathogenic, prevalence

## Abstract

**Aim:**

Annual reports have shown that 20% of fruits and vegetables produced are lost to spoilage. This study was undertaken to isolate and identify fungi that are associated with spoilt fruits commonly sold in Gwagwalada market, Abuja, and recommend appropriate control measure.

**Materials and Methods:**

The study was conducted in Gwagwalada metropolis, Gwagwalada Area Council of the Federal Capital Territory, Abuja, Nigeria. A total of 100 spoilt fruits which include pawpaw (*Carica papaya*), orange (*Citrus sinensis*), tomato (*Lycopersicon esculentum*), pineapple (*Ananas comosus*), and watermelon (*Citrullus vulgaris*) were purchased and examined for the presence of fungal organisms using standard methods. The data collected were analyzed using simple descriptive statistics (frequency and mean) and analysis of variance (p<0.05).

**Results:**

*Aspergillus niger* had the highest occurrence in pineapple, watermelon, oranges, pawpaw, and tomatoes with a frequency of 38%. *Fusarium avenaceum* followed with the frequency of occurrence of 31% in fruits such as pineapple, watermelon, oranges, pawpaw, and tomatoes while *Penicillium digitatum* and *Rhizopus stolonifer* had the least frequency of 4% each in tomato; and orange and tomato, respectively. Other fungal species were identified as yeast (*Saccharomyces* species) (10%), *Fusarium solani* (8%), and *Aspergillus flavus* (5%). The highest prevalence rate was 70% of *A. niger* from orange followed by *F. avenaceum* of which 65% isolates were recovered from pawpaw. Other fungal organisms such as yeast (*Saccharomyces* species), *P. digitatum* and *R. stolonifer* were isolated with varying prevalence (40%, 20%, and 5%) from watermelon, tomato, and orange, respectively. However, there was no significant difference in the fungal load of the various fruits studied (analysis of variance=478.2857, p<0.05, F=4.680067 and df=34).

**Conclusion:**

The pathogenic fungi species associated with fruits spoilage in this study are of economical and public health significance. *A. niger* causes black mold in certain fruits and vegetables. Some strains of *A. niger* have been reported to produce potent mycotoxins called ochratoxins that can be harmful to human beings and animals. Care should be taken during handling of these fruits and improved technology based preservation methods are suggested to enhance the keeping quality of fruits.

## Introduction

Fruits are the comestible part of mature ovary of flowering plants which are normally eaten raw [1]. Fruit also includes many structures that are not commonly called fruits such as bean pods, corn kernels, tomatoes, and wheat grains [1]. The importance of fruit in human nutrition cannot be overestimated as it provides essential growth factors such as vitamins and minerals necessary for proper body metabolism [2]. Humans and many animals have become dependent on fruits as a source of food [3].

However, fruits are easily spoilt and usually have active metabolism during the storage stage [4]. The high concentration of various sugars, minerals, vitamins, amino acids, and low pH also enhances the successful growth and survival of various parasitic and saprophytic forms of fungi [5]. Annual reports have shown that 20% of fruits and vegetables produced are lost to spoilage [6,7], especially during post-harvest stages [4]. This has been associated with spoilage fungi which can be toxigenic or pathogenic [8]. Toxin-producing fungi have been identified and isolated from spoilt fruits by previous researchers [2]. Pathogenic fungi have been reported in cases of infections or allergies [9]. *Aspergillus* spp. produces mycotoxins and other toxic metabolites which can be harmful to humans and animals globally [10,11].

Micro-organisms, especially fungi, are known to destroy fruits, thereby reducing the quantity for consumption and the profits obtained from sales of fruits. There is need to identify these micro-organisms especially those that are pathogenic to humans so as to reduce the risk of contamination and infection arising from handling and consumption of fruits. Therefore, this study was undertaken to isolate and identify fungi associated with spoilt fruits commonly sold in Gwagwalada market, Abuja, and recommend appropriate control measure.

## Materials and Methods

### Ethical approval

The research outline was conducted as approved by the Faculty of Science and Department of Biological Sciences Seminar Committee, University of Abuja, FCT, Nigeria.

### Study area

The study was conducted in Gwagwalada metropolis, Gwagwalada Area Council of the Federal Capital Territory (FCT), Abuja, Nigeria. Gwagwalada is one of the six Area Councils of the FCT, Abuja; alongside Abaji, Kuje, Bwari, Kwali, and Abuja Municipal Area Council. Gwagwalada covers an estimated land mass of 1043 km^2^ and a population of 157,770 during the 2006 census [12], where the University of Abuja is located. Gwagwalada is located on geographical coordinates of 8°56′29″ North, 7°5′31″ East (three-dimensional Google Earth). The area is characterized by two seasons - wet and dry seasons. The dry season lasts between May and October with a unimodal peak of rainfall in August. Gwagwalada Area Council is extremely hot in terms of temperature with a mean daily temperature of 31°C [13].

### Fruit materials

Five types of fruits which comprise 20 each of pawpaw (*Carica papaya*), orange (*Citrus sinensis*), tomato (*Lycopersicon esculentum*), pineapple (*Ananas comosus*), and watermelon (*Citrullus vulgaris*) were purchased from Gwagwalada market and transported to Mycology Laboratory, Department of Biological Sciences, University of Abuja, Abuja for fungal analysis.

### Isolation of fungi

A total of 100 randomly selected spoilt fruits and another 100 healthy looking fruits were examined. The fruits were cut into small segments (3 mm in diameter) with a sterilized blade, surface sterilized in 1% hypochlorite for 2 min, plated on Sabouraud dextrose agar (SDA) aseptically and then incubated at 28°C for 5 days.

A pure culture was obtained and maintained by sub-culturing each of the different colonies that emerged onto the SDA plates and incubating at 28°C for 5 days.

As a control, each of the healthy fruits was sterilized with 75% ethanol. The fruits were cut into small segments (3 mm in diameter) with a sterile blade, placed on SDA and then incubated at 28°C for 5 days.

### Identification of isolated fungi

The fungal isolates were identified using cultural and morphological features such as colony growth pattern, conidial morphology, and pigmentation [14]. The technique of Oyeleke and Manga [15] was also adopted for the identification of the isolated fungi using cotton blue in lactophenol stain. The identification was achieved by placing a drop of the stain on clean slide with the aid of a mounting needle, where a small portion of the aerial mycelia from the representative fungi cultures was removed and placed in a drop of lactophenol. The mycelium was well spread on the slide with the needle. A cover slip was gently placed with little pressure to eliminate air bubbles. The slide was then mounted and viewed under the light microscope with ×10 and ×40 objective lenses. The morphological characteristics and appearance of the fungal organisms seen were identified in accordance with Adebayo-Tayo *et* *al*. [16], Onuorah *et al*. [17], Klich [18], Samson and Varga [19].

### Statistical analysis

Statistical Package for the Social Science was used for the data analysis. The fungi isolated were recorded as frequency and prevalence. Analysis of variance (ANOVA) was used to compute and arrived at statistical decision and p<0.05 was considered significant.

## Results

Our research conducted to screen vended fruits in Gwagwalada for the presence of pathogenic fungi yielded array of fungal species.

Table-1 shows the frequency of occurrence of fungi in the various fruits. *Aspergillus niger* had the highest occurrence in pineapple, watermelon, oranges, pawpaw, and tomatoes with a frequency of 38%. *Fusarium avenaceum* followed with the frequency of occurrence of 31% in fruits such as pineapple, watermelon, oranges, pawpaw, and tomatoes while *Penicillium digitatum* and *R. stolonifer* had the least frequency of 4% each in tomato; and orange and tomato, respectively. Other fungal species were identified as yeast (*Saccharomyces* species) (10%), *F. solani* (8%) and *Aspergillus flavus* (5%).

**Table-1:** Frequency of occurrence of fungal species.

Fungi isolates	Fruits infected	Frequency of occurrence (%)
*Aspergillus niger*	Pineapple, watermelon, orange, pawpaw, tomato	38
*Fusarium avenaceum*	Pineapple, watermelon, orange, pawpaw, tomato	31
Yeast (*Saccharomyces* spp.)	Watermelon, orange and pawpaw	10
*Fusarium solani*	Pineapple, watermelon, pawpaw and tomato	8
*Aspergillus flavus*	Tomato	5
*Penicillium digitatum*	Tomato	4
*Rhizopus stolonifer*	Orange and tomato	4

Table-2 shows the prevalence and distribution of fungi isolates associated with 100 spoilt fruits vended in Gwagwalada. The highest prevalence rate was 70% of *A. niger* from orange followed by *F. avenaceum* of which 65% isolates were recovered from pawpaw. Other fungal organisms - such as yeast (*Saccharomyces* species), *P. digitatum*, and *R. stolonifer* - were isolated with varying prevalence (40%, 20% and 5%) from watermelon, Tomato and Orange, respectively.

**Table-2:** Prevalence and distribution of fungi associated with spoilt fruits vended in Gwagwalada.

Fungal isolates	Fruit type				

	Pineapple (n=20)	Tomato (n=20)	Watermelon (n=20)	Orange (n=20)	Pawpaw (n=20)
*Aspergillus niger*	10 (50)	3 (15)	7 (35)	14 (70)	4 (20)
*Fusarium avenaceum*	7 (35)	3 (15)	4 (20)	4 (20)	13 (65)
Yeast (*Saccharomyces* spp.)	0 (0)	0 (0)	8 (40)	1 (5)	1 (5)
*Fusarium solani*	3 (15)	2 (10)	1 (5)	0 (0)	2 (10)
*Aspergillus flavus*	0 (0)	5 (25)	0 (0)	0 (0)	0 (0)
*Penicillium digitatum*	0 (0)	4 (20)	0 (0)	0 (0)	0 (0)
*Rhizopus stolonifer*	0 (0)	3 (15)	0 (0)	1 (5)	0 (0)

n: Number of each fruit sampled/tested

However, there was no significant difference in the fungal load of the various fruits studied (ANOVA=478.2857, p<0.05, F=4.680067 and df=34).

No growth was observed in the control group on SDA at 28°C for 5 days.

## Discussion

The isolation and distribution of fungi in spoilt fruits in Gwagwalada is a novel discovery which exposed array of fungi which are pathogenic to man and animals. The identified fungal organisms associated with spoilt fruits in the study area include *A. niger, A. flavus, F. solani*, *F. avenaceum, P. digitatum, R. stolonifer*, and yeast (*Saccharomyces* species) suggesting that these fungal organisms could be responsible for the fruit spoilage. This finding is in conformity with previous works of Baiyewu *et al*. [20] and Chukwuka *et al*. [21] which reported isolation of *A. niger*, *F. avenaceum, R. stolonifer* and yeast from pawpaw in Nigeria.

Previous literature indicates that processes such as harvesting, storing, packing and transporting, fruits may encounter physical injury that increases post-harvest loss and the possibility of fungal contamination. In addition, the problem can be enhanced from poor management of fruits in Gwagwalada market [22]. Market conditions that favor contamination can be worsened by poor hygiene of the vendors, using microbial unsafe container poor handling practice and poor environmental conditions such as sanitarily unsafe marketing environment. The consequence of the problems could be increased loss of fruit due to microbial spoilage and the existence of some human pathogens [22,23].

Out of fungi isolated in this study, *A. niger* which causes a disease called black mold on certain fruits and vegetables and produces potent mycotoxins called ochratoxins that can be harmful to human beings and animals had the highest occurrence (38%) followed by *F. avenaceum* (31%) while *P. digitatum* (4%) and *R. stolonifer* (4%) had the lowest frequency of occurrence. However, Tafinta *et al*. [14] reported a frequency of occurrence of 36%, 25%, 22%, and 17% for *R. stolonifer*, *A. flavus, A*. *fumigatus*, and *A. niger*, respectively, from sweet oranges. These differences could be attributed to number and type fruits examined in both studies.

Most of the fungal organisms isolated in this study play a pivotal role in the deterioration of food and feed systems and some of them are also able to produce toxic compounds for humans and animals. The mycotoxins produced by these fungi can cause serious health hazards including cancerogenic, immunotoxic, teratogenic, neurotoxic, nephrotoxic and hepatotoxic effects, and Kashin-Beck disease [24]. *F. avenaceum* is well-known for causing ear blight and root rot of cereals, blights of plant species within genera as diverse as *Pinus* and *Eustoma*, as well as post-harvest storage rot of numerous crops, including potato, broccoli, apple and rutabaga. *F. avenaceum* has also been described as an endophyte, and an opportunistic pathogen of animals [25-28]. *P. digitatum* causes a destructive fruit rot of citrus. It is generally considered the most important agent of post-harvest spoilage in the most citrus species. Early symptoms include a soft water-soaked area on the peel, followed by the development of a circular colony of white mold, up to 4 cm diameter after 24-36 h at 24°C. *Penicillium* species are common fungi in the environment and are often considered non-pathogenic to humans [29]. R. *stolonifer* is a significant agent of fruit disease. It is a threadlike mold and a heterotrophic species; it depends on sugar or starch for its source of carbon substances for food. It uses food matter, mostly soft fruits, like grapes or strawberries, as a food source for growth, nutrition and reproduction [30].

In this study, fungal organisms were isolated from pineapple, watermelon, pawpaw, orange and tomato. *A. niger* and *F. avenaceum* were more widespread among all the spoilt fruits examined followed by *F. solani* and yeast (*Saccharomyces* species). *P. digitatum* and *A. flavus* were isolated from only tomato. Similar findings on the isolation fungal pathogens from fruits stored and sold in the market have been reported by earlier researchers [31]. Bali *et al*. [32] stated that *A. niger* was the cause of post-harvest spoilage in sweet orange and acid lime at field. Okereke *et al*. [33] reported that *A. niger*, *Alternaria* species., *Botryodiplodia theobromae*, and *Colletotrichum gloeosporioides* were isolated from the spoilt mangoes. Chukwuka *et al*. [21] implicated *Rhizopus* *nigricans, A. flavus, A. niger, Fusarium* spp., and *Mucor* spp. in pawpaw fruit spoilage from a farm in Oyo state, Nigeria.

Fungal pathogens are causing losses of marke table quality and hygiene of fruits, resulting in major economic problem in Nigeria and the world at large. Fruit spoilage can be prevented using physical [34] and chemical methods [35], but no efficient strategy has been proposed so far to reduce the microbial growth ensuring public health safety. Lactic acid Bacteria (LAB) can play a vital role as natural preservatives. The protection of fruits or fruit products using LAB is mainly because of the production of antifungal compounds such as carboxylic acids, fatty acids, ethanol, carbon dioxide, hydrogen peroxide, and bacteriocins [24].

The control experiment showed no fungal growth on SDA after healthy fruits were sterilized with 75% ethanol indicating that the isolated fungi were introduced post-harvest from farms through fruit vendors and finally to consumers [4]. Fresh fruits recently have been identified as a significant source of plant and human pathogens and chemical contaminants that pose a potential threat to human health worldwide. Because it is likely to be eaten raw by scavenging animals especially ruminants, humans also stands risk of getting infected with pathogenic fungi from fruits and vegetables as a results of poor processing methods. More so, fresh fruits pose potential food safety hazard and poor type of microbiologically lethal processing regime could lead to potential food safety problems. Poor handling can damage fresh fruits, rendering their products susceptible to the growth or survival of spoilage and pathogenic microorganisms [22].

## Conclusions

This study has shown that *A. niger, A. flavus, F. solani*, *F. avenaceum, P. digitatum, R. stolonifer* and yeast (*Saccharomyces* species) were isolated from spoilt pineapple, watermelon, pawpaw, orange, and tomato. However, some fruits such as pineapple, watermelon, oranges, and pawpaw are free from contamination with fungi such as *A. flavus, P. digitatum*, and *R. stolonifer*. These pathogenic fungi species associated with fruits spoilage are of economical and public health significance. Care should be taken during handling of these fruits, technology based modern preservative methods such as pasteurization, vacuum packing, radiation, pulsed electric field electroporation, high-pressure food preservation, and bio preservation are suggested to enhance the keeping quality of fruits.

## Authors’ Contributions

SM designed the study and contributed to the preparation of the manuscript. Laboratory work was done by RO. GRO prepared the manuscript, analyzed the data and corresponded the authorship. HOKO revised the manuscript and further assisted in the discussion and manuscript review. All authors read and approved the final manuscript for publication.
